# Developing a human iPSC-derived three-dimensional myelin spheroid platform for modeling myelin diseases

**DOI:** 10.1016/j.isci.2023.108037

**Published:** 2023-09-25

**Authors:** Lizhao Feng, Jianfei Chao, Mingzi Zhang, Elizabeth Pacquing, Weidong Hu, Yanhong Shi

**Affiliations:** 1 Department of Neurodegenerative Diseases, Beckman Research Institute of City of Hope, 1500 E. Duarte Road, Duarte, CA 91010, USA; 2 Department of Molecular Imaging and Therapy, Beckman Research Institute of City of Hope, 1500 E. Duarte Road, Duarte, CA 91010, USA; 3 Oujiang Laboratory, Zhejiang Lab for Regenerative Medicine, Vision and Brain Health, Wenzhou, Zhejiang 325000, China

**Keywords:** Molecular biology, Cellular biology, Neuroscience, Techniques in neuroscience

## Abstract

Myelin defects cause a collection of myelin disorders in the brain. The lack of human models has limited us from better understanding pathological mechanisms of myelin diseases. While human induced pluripotent stem cell (hiPSC)-derived spheroids or organoids have been used to study brain development and disorders, it has been difficult to recapitulate mature myelination in these structures. Here, we have developed a method to generate three-dimensional (3D) myelin spheroids from hiPSCs in a robust and reproducible manner. Using this method, we generated myelin spheroids from patient iPSCs to model Canavan disease (CD), a demyelinating disorder. By using CD patient iPSC-derived myelin spheroids treated with N-acetyl-aspartate (NAA), we were able to recapitulate key pathological features of the disease and show that high-level NAA is sufficient to induce toxicity on myelin sheaths. Our study has established a 3D human cellular platform to model human myelin diseases for mechanistic studies and drug discovery.

## Highlights

• Develop a method to generate myelin spheroids
• Generate myelin spheroids in a simple and standardized process
• Myelin spheroids contain compact myelin sheaths with paranodes
• Patient myelin spheroids can recapitulate key pathologies of Canavan disease

## Graphical abstract

**Figure:**
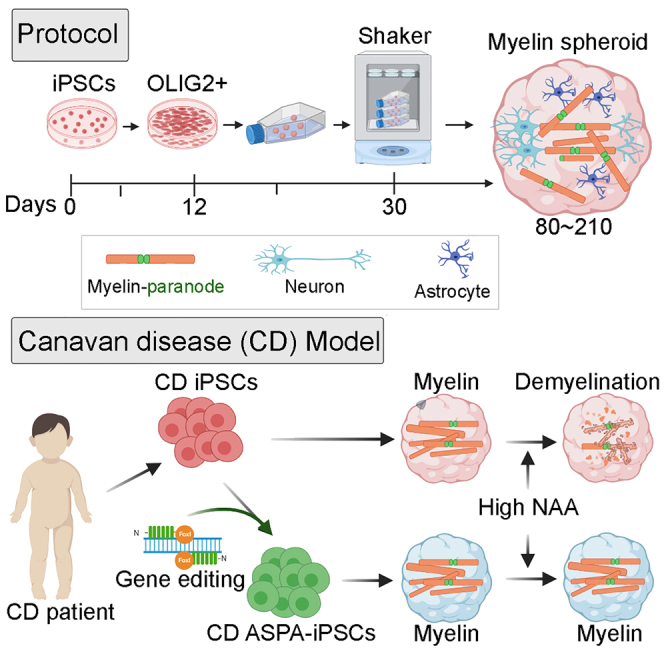

## Introduction

Myelin disorders in the central nervous system (CNS) form a large and growing list of neurological diseases in humans, ranging from the most common myelin disease, multiple sclerosis (MS), to rare genetic disorders, leukodystrophies.^1^ Besides myelin loss caused by myelin disorders, defects in myelination and remyelination have also been observed in other disorders, such as Alzheimer’s disease and acute CNS injuries.^2^^,^^3^ Rodent models have been used to model myelin dysfunction and provided great insights into pathological mechanisms underlying these disorders.^1^ However, species difference has prevented comprehensive modeling of complex myelination diseases, such as MS. The complexity of *in vivo* environment also limits the ability to identify key factor(s) responsible for myelin defects using rodent models alone. It is essential to have a suitable *in vitro* human myelin model to complement rodent models for studying pathological mechanisms underlying myelin diseases.

Human induced pluripotent stem cells (hiPSCs) provide a powerful platform for us to model human diseases,^4^^,^^5^ especially brain disorders for which human cells and tissues are not readily accessible.^6^^,^^7^^,^^8^ hiPSC-derived three-dimensional (3D) spheroids or organoids have been used to study brain development and disorders in our group and others.^9^^,^^10^^,^^11^^,^^12^^,^^13^^,^^14^^,^^15^^,^^16^^,^^17^^,^^18^^,^^19^ However, it has been difficult to recapitulate mature myelination in these 3D structures. To address this limitation, methods have been developed to generate 3D oligospheres with mature oligodendrocytes and myelination.^20^^,^^21^^,^^22^^,^^23^^,^^24^^,^^25^ However, spheroids generated from these protocols showed limited compact myelination. In a more recent study, a protocol was developed to generate myelinoids with widespread compact myelination and myelinated axons on cell culture inserts.^26^ How to simplify the protocol to allow easier manipulation and wider applications remains a tempting question.

Canavan disease (CD) is one of the classical demyelinating diseases that belong to leukodystrophies.^1^^,^^27^ CD is caused by genetic mutation in the aspartoacylase (*ASPA*) gene, which encodes a metabolic enzyme synthesized by oligodendrocytes in the brain.^28^ ASPA hydrolyzes N-acetyl-aspartate (NAA) generated by neurons to acetate and aspartic acid. The lack of ASPA enzyme activity causes accumulation of NAA, myelin vacuolation and demyelination.^1^^,^^29^^,^^30^ There are two hypotheses for the mechanism of demyelination in CD: “NAA toxicity”, meaning accumulated NAA-induced toxicity, or “oligodendroglial starvation” due to a decrease in acetate level-caused loss of myelin-associated lipids.^31^ Which mechanism underlies myelination defects in CD remains to be understood.

In this study, we developed a protocol to differentiate hiPSCs into 3D myelin spheroids to model oligodendrocyte genesis and myelination. Our approach generated mature oligodendrocytes from OPCs alongside neurons and astrocytes following the developmental process. Myelin produced from oligodendrocytes wrapped axons and formed compact myelin sheaths with paranodes. Moreover, we were able to model CD using CD patient iPSC-derived myelin spheroids and recapitulate key pathological features of the disease.

## Results

### Generation and characterization of hiPSC-derived oligodendroglial spheroids

To produce myelin in 3D spheroids derived from hiPSCs, we adapted a widely used oligodendrocyte progenitor cell (OPC) differentiation protocol to generate mature oligodendrocytes, along with astrocytes and neurons that are needed for oligodendrocyte maturation and myelination.^32^^,^^33^ IMR90 (I90) iPSCs were derived from human I90 fibroblasts and used to set up the protocol. Briefly, hiPSCs were dissociated into single cells and seeded onto plates and serially induced by neural and glial lineage induction media.^32^^,^^33^^,^^34^ Cells were then detached for spheroid formation and cultured in flasks until spheroids were collected. The glial lineage induction medium, OPC expansion medium, and oligodendrocyte maturation medium were added sequentially to induce myelination. The spheroids were collected and characterized every two weeks from day 30 (∼ week 4).

**Figure 1:**
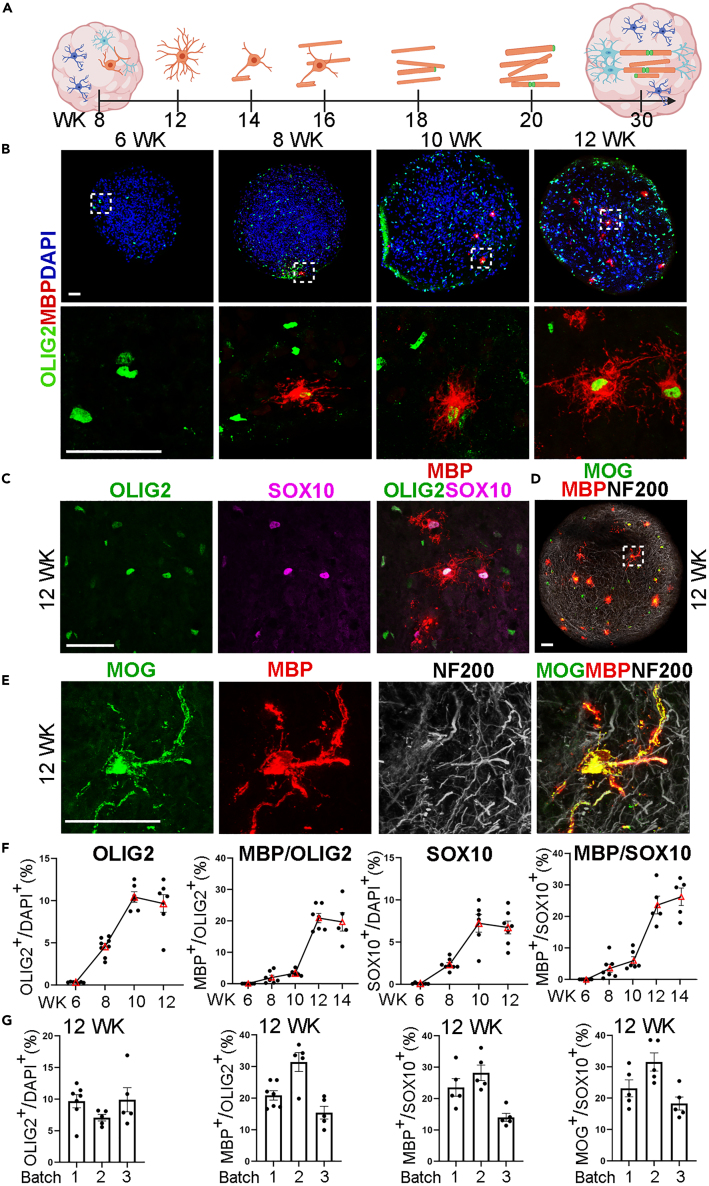
Generation and characterization of hiPSC-derived oligodendroglial spheroids (A) Schematic of myelin spheroid generation and myelin formation. The oligodendrocyte and myelin sheaths are shown in brown; paranodes in green; astrocytes in blue, and neurons in cyan. (B) Immunostaining of week 6 to week 12 spheroids for the oligodendrocyte lineage marker OLIG2 and the mature oligodendrocyte marker MBP. Scale bar: 50 μm. (C) Oligodendrocytes co-expressed oligodendroglial markers OLIG2, SOX10, and MBP. Scale bar: 50 μm. (D, E) Co-staining of spheroids for oligodendroglial markers MOG and MBP with the neuron axon marker NF200. Myelination starts to be observed in spheroids at week 12 in panel E. Scale bar: 50 μm for d and e. (F) The percentage of the OLIG2^+^ and SOX10^+^ oligodendroglial cells in week 6 to week 12 spheroids, and the percentage of the MBP^+^ oligodendrocytes (MBP^+/^SOX10^+^, MBP^+/^OLIG2^+^) in week 6 to week 14 spheroids. (G) The percentage of the OLIG2^+^ oligodendroglial cells, the MBP^+^ oligodendrocytes (MBP^+/^SOX10^+^, MBP^+/^OLIG2^+^), and the MOG^+^ oligodendrocytes (MOG^+/^SOX10^+^) in 3 batches of I90 spheroids. The week 12 data of OLIG2, MBP/OLG2, and MBP/SOX10 in panel F are shown as batch 1 results in panel G. The average data of 3 sections from each spheroid were taken and plotted as one dot. n ≥ 5 spheroids for each group. Error bars are SE of the mean for panels (F and G).

Mature neurons expressing NeuN and MAP2 and mature astrocytes expressing GFAP and SOX9 were found in I90 spheroids 30 days after differentiation (Figures S1A and S1C). Both the NeuN^+^ neurons and SOX9^+^ astrocytes exhibited at high density in early stage spheroids (Figures S1B and S1D). These two cell types have been shown to promote oligodendrocyte maturation and myelination.^35^^,^^36^^,^^37^^,^^38^ Six weeks after induction, I90 spheroids were collected every two weeks to track myelination. At week 6, we detected OLIG2-positive (OLIG2^+^) cells but not myelin basic protein (MBP)-positive (MBP^+^) cells in the spheroids (Figure 1B). The MBP^+^ oligodendrocytes emerged around week 8 (Figures 1A and 1B). The MBP^+^ cells were also positive for the oligodendrocyte lineage markers OLIG2 and SOX10 (Figure 1C). The identity of mature oligodendrocytes in spheroids was confirmed by co-expression of MBP and myelin oligodendrocyte glycoprotein (MOG) along with the neurofilament 200 (NF200)-positive neuronal axons (Figures 1D and 1E). The proportion of the OLIG2^+^ and SOX10^+^ oligodendroglial lineage cells increased from week 6 to week 10, followed by an increase in the percentage of the MBP^+^OLIG2^+^ or MBP^+^SOX10^+^ mature oligodendrocytes from week 8 to week 12 (Figure 1F). The maturation of oligodendrocytes was revealed by the shift of cell morphology from a less branched to a highly branched configuration. The emergence of line-shaped and tube-shaped MBP signals along NF200^+^ neuronal axons around week 12 (Figure 1E) suggests that the appearance of MBP-positive-membrane-contacting axons and early myelination was initiated from week 12. The OLIG2^+^ and/or SOX10^+^ oligodendroglial lineage cells, and MBP^+^ and/or MOG^+^ mature oligodendrocytes were consistently generated in different batches of I90-iPSC-derived spheroids (Figure 1G) and in spheroids derived from two other iPSC lines, CD#68 and CD#59, using this protocol (Figure S2). Taken together, the protocol we developed in this study generated spheroids containing mature oligodendrocytes with supporting neurons and astrocytes.

### Myelination by hiPSC-derived oligodendrocytes in myelin spheroids

**Figure 2:**
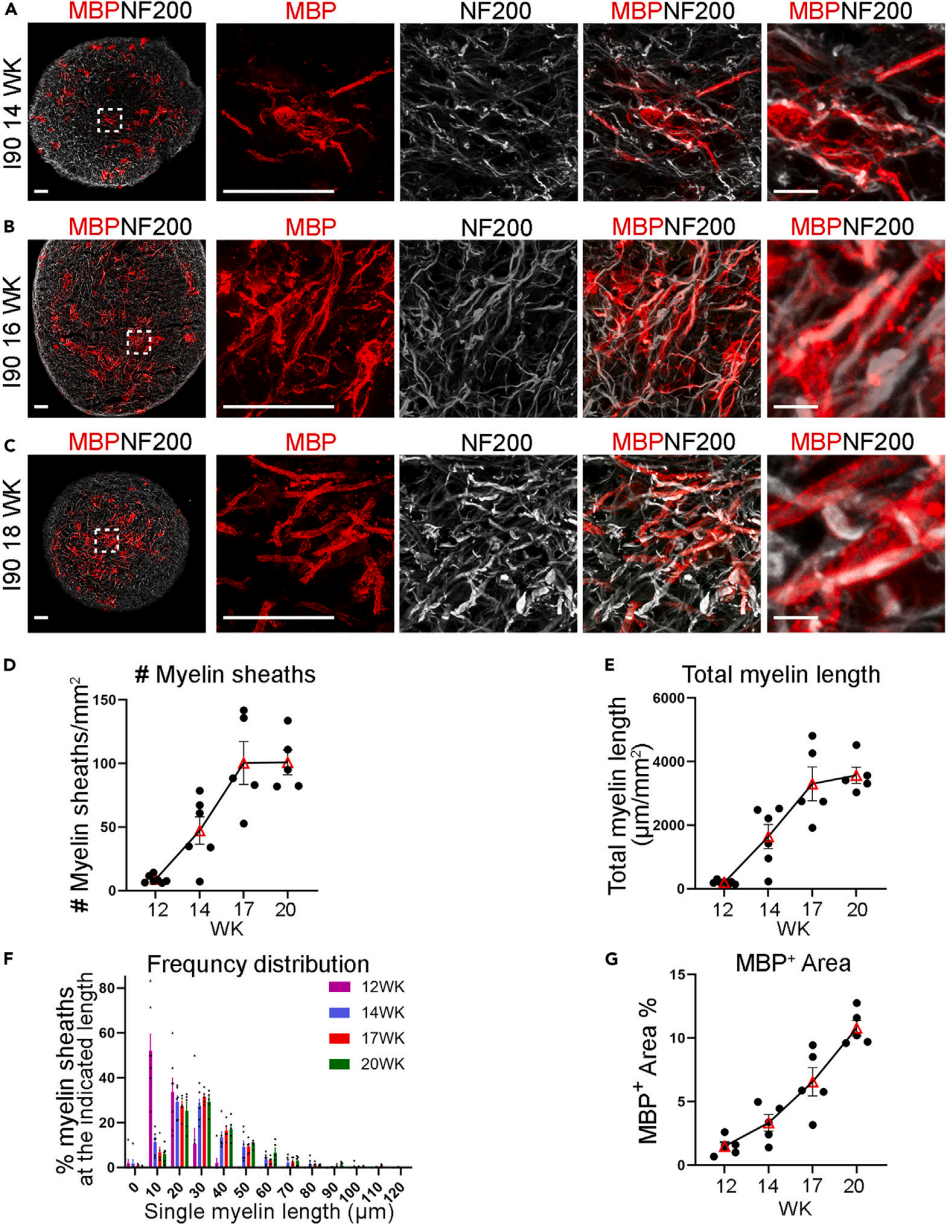
Myelin formation in myelin spheroids (A–C) Immunostaining of myelin sheaths for MBP and NF200 at week 14, week 16 and week 18. Scale bar: 50 μm for the left four panels; 10 μm for the rightmost panel. (D, E) The number (D) and total length (E) of the MBP^+^ myelin sheaths were increased in spheroids from week 12 to week 20. n = 7 spheroids for week 12, n = 6 spheroids for week 14, and n = 5 for week 17 and week 20, respectively. The average data of 3 sections from each spheroid were taken and plotted as one dot. (F) The frequency distribution of the MBP^+^ single myelin sheaths showed myelin sheath growth from week 12 to week 20. n = 61 myelin sheaths from 7 spheroids for week 12, n = 262 myelin sheaths from 6 spheroids for week 14, n = 603 myelin sheaths from 5 spheroids for week 17, n = 577 myelin sheaths from 5 spheroids for week 20. (G) The percentage of MBP^+^ area increased in spheroids from week 12 to week 20. The average data of 3 sections from each spheroid were taken and plotted as one dot. n = 5 spheroids for each group. Error bars are SE of the mean for panels (D–G).

The MBP^+^ processes from mature oligodendrocytes began to contact axons from week 12 post-differentiation, although we could only find a few myelin-sheath-like structures at this stage (Figure 1E). To further track myelination, we stained I90 myelin spheroid sections with MBP and NF200 from week 14 to week 18 post-differentiation. Additional short myelin sheaths with tube-like structures were formed, and more MBP-positive processes closely aligned with axons at week 14 (Figure 2A). From week 16 to week 18, myelin sheaths showed evident growth in length (Figures 2B and 2C). The myelin sheaths were manually traced, and the length was measured (Figure S3A). Both the number and the total length of myelin sheaths increased dramatically from week 17 to week 20 (Figures 2D and 2E). An increase of the myelin sheath length with time was observed from week 12 to week 20 (Figure 2F). The MBP^+^ area also increased from week 12 to week 20 (Figure 2G). The myelin-sheath structure was also detected in CD#68 myelin spheroids (Figures S3B and S3C). The number and the total length of myelin sheaths and the MBP^+^ area in CD#68 myelin spheroids increased with time from week 12 to week 20 (Figures S3D–S3G). The myelin-sheath structure was detected in multiple batches of CD#68 myelin spheroids (Figures S3H and S3I), supporting the consistency of the protocol.

### Paranode formation in myelin spheroids

**Figure 3:**
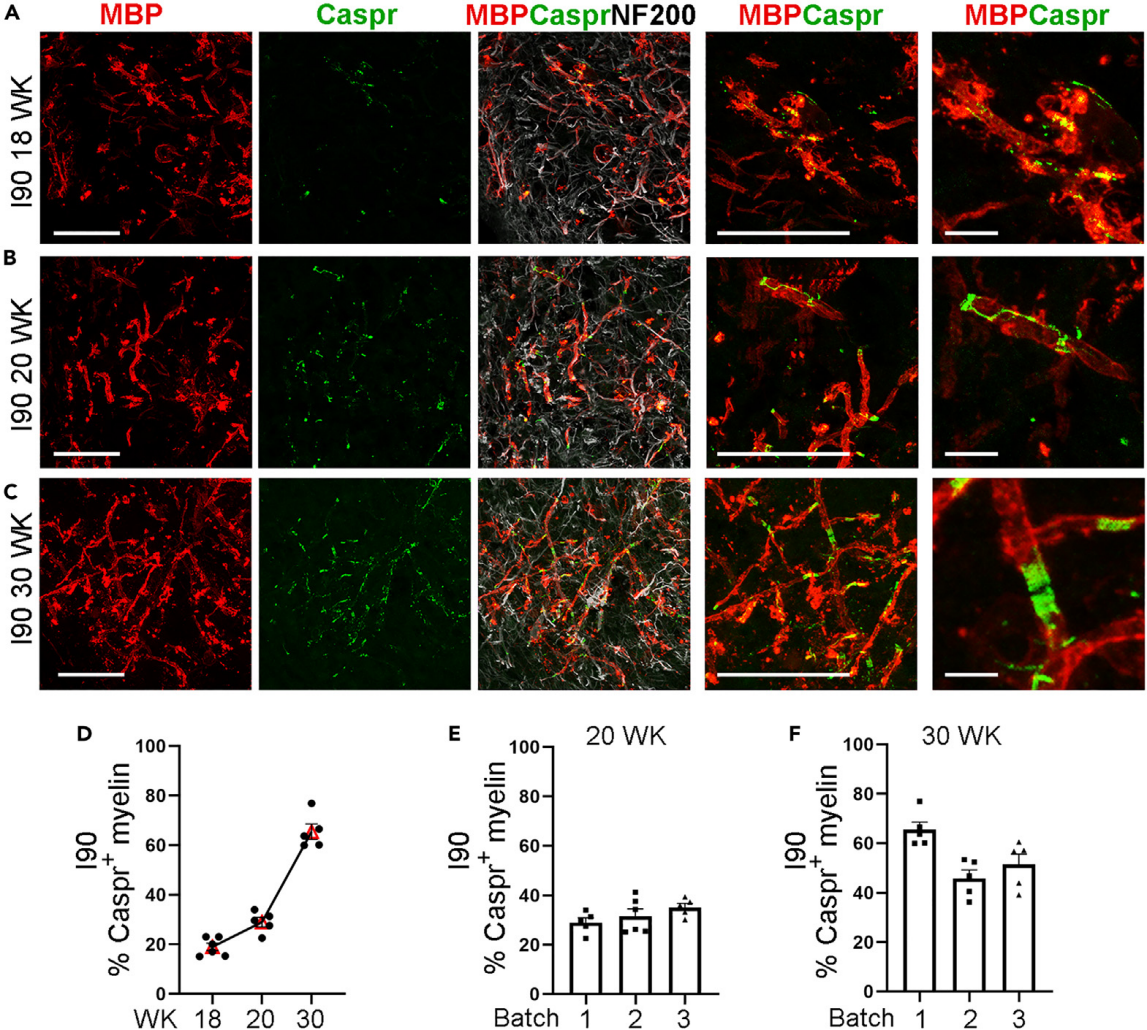
Paranode formation in myelin spheroids (A) Schematic of myelin spheroid generation. (B–D) Immunostaining of spheroids for MBP and the paranodal marker Caspr at week 18 (B), week 20 (C) and week 30 (D). Caspr was observed as early as week 18 with low density (B). At week 20, Caspr was observed to be concentrated with a long spiral-like tail (C). At week 30, concentrated Caspr and paired paranodes are shown. Scale bar: 50 μm for the left four panels; 10 μm for the rightmost panel in panels A to C. (D) The percentage of myelin sheaths with the Caspr^+^ ends in total myelin sheaths from week 18 to week 30. n = 6 spheroids for week 18, n = 5 spheroids for week 20 and week 30, respectively. (E) The percentage of myelin sheaths with the Caspr^+^ ends in total myelin sheaths from three different differentiation batches on week 20. n = 5 spheroids for batch 1, n = 6 spheroids for batch 2, and n = 5 spheroids for batch 3. (F) The percentage of myelin sheaths with the Caspr^+^ ends in total myelin sheaths from three different differentiation batches on week 30. n = 5 spheroids for each batch. The data from week 20 and week 30 in panel D are shown as batch 1 result in panels E and F, respectively. The average data of 3 sections from each spheroid were taken and plotted as one dot. Error bars are SE of the mean for panels (D–F).

Because nodes of Ranvier, flanked by paranodal junctions, play a critical role in the fast saltatory conduction of action potentials along myelinated axon,^39^ we stained myelin spheroids with the paranodal marker Caspr to check node formation. The expression of Caspr was first observed at week 18 in I90 myelin spheroids (Figure 3A). Caspr was detected along the edge of the myelin membranes and the axons at week 18 as revealed by immunostaining (Figure 3A). With further maturation of the myelin sheaths, Caspr was observed to be concentrated in the paranodal region with a long spiral-like tail at week 20 (Figure 3B). At week 30, the expression of Caspr was restricted to paranodes (Figure 3C), indicating that the myelin sheaths are fully mature. The presence of the concentrated Caspr and paranodes suggests the formation of the nodes of Ranvier. The percentage of myelin sheaths with the Caspr end increased from about 18% on week 18 to about 65% on week 30 (Figure 3D). The I90 myelin spheroids from three different batches showed comparable percentage of Caspr^+^ myelin sheaths (Figures 3E and 3F). Concentrated Caspr signals in the paranodal region were also observed in multiple batches of CD#68 myelin spheroids (Figure S3J).

### The ultrastructure of myelin in myelin spheroids

**Figure 4:**
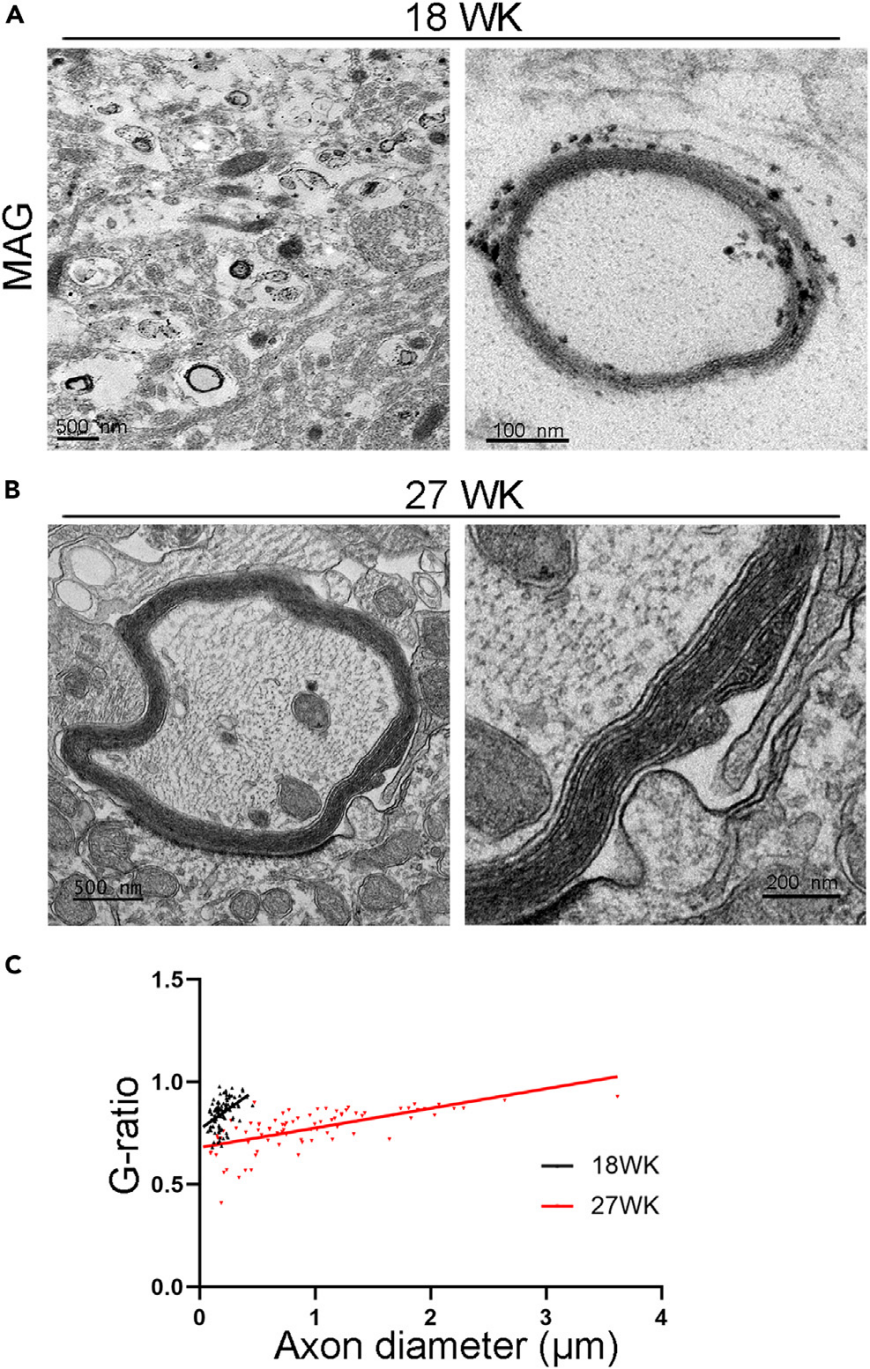
The ultrastructure of myelin sheaths in myelin spheroids (A) Immunogold staining of I90 myelin spheroids for the myelin marker MAG at week 18. The black dots located on the myelin sheath were MAG-positive signals. Scale bar: 500 nm for the left panel, 100 nm for the right panel. (B) Compact myelin sheaths with multiple layers are shown by transmission electron microscopy (TEM). The scale bar is shown in the images. Scale bar: 500 nm for the left panel, 200 nm for the right panel. (C) Quantitation of the g-ratio as the function of the axon diameter from week 18 and week 27 with the regression lines. The g-ratio of one myelin sheath is shown as one dot in the graph. n = 108 myelin sheaths from 3 spheroids for week 18 and n = 82 myelin sheaths from 3 spheroids for week 27.

We performed transmission electron microscopy combined with immunogold staining to identify the ultrastructure of myelin. At week 18, multiple early-stage myelin sheaths were found with thin sheaths and few layers (Figure 4A). Staining for MAG by immunogold staining confirmed that the structures are myelin sheaths. After further culture, we found mature myelin sheaths with well-organized compact and multi-layer structure by week 27 (Figure 4B). Moreover, the analysis of g-ratio, the ratio between the inner diameter (axon) and the outer diameter of the myelin sheaths, revealed increased maturation of myelin sheaths from week 18 (mean g-ratio = 0.85) to week 27 (mean g-ratio = 0.77) (Figure 4C), the latter of which is within the range of g-ratio in human brains (mean g-ratio = 0.6–0.8).^40^

### Patient iPSC-derived myelin spheroids recapitulate key pathologies of CD

Next, we asked whether our myelin spheroids can be used to model myelin disorders. CD is a fatal leukodystrophy caused by mutation in the *ASPA* gene, which leads to a deficiency in the ASPA enzymatic activity. The deficiency of the ASPA activity causes accumulation of NAA in the brain, accompanied by spongy degeneration characterized by vacuolation and demyelination.^1^^,^^28^

**Figure 5:**
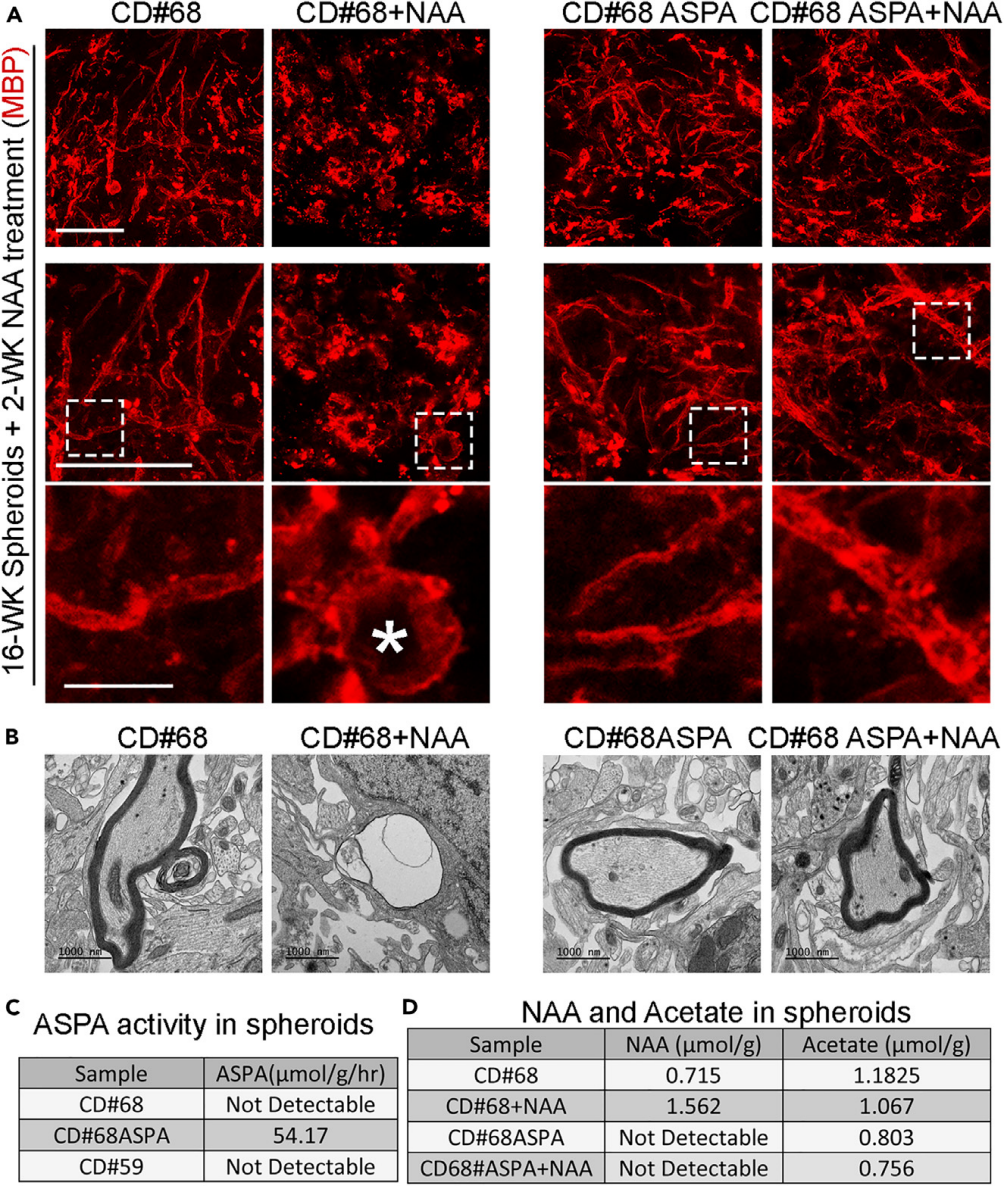
CD patient iPSC-derived myelin spheroids recapitulate myelin pathologies after NAA treatment (A) Immunostaining of CD-myelin spheroids with or without NAA treatment for MBP. CD#68 hiPSC-derived spheroids formed myelin sheaths normally *in vitro*. Myelin damage and vacuolation were observed in CD#68 spheroids after NAA treatment. Images were enlarged sequentially from the top to the bottom. The “∗” in the CD#68+NAA sample indicates an example of scattered myelin sheath. Scale bar: 50 μm for the top and middle rows; 10 μm for the bottom row. (B) TEM images showing the ultrastructure of myelin sheaths in CD#68 or CD#68 ASPA myelin spheroids with or without NAA treatment. (C) The ASPA activity of CD#68, CD#68 ASPA, and CD#59 myelin spheroids. (D) The concentration of NAA and acetate in CD#68 or CD#68 ASPA spheroids with or without NAA treatment. ND, not detectable.

We used CD#59 and CD#68 iPSCs derived from fibroblasts of two CD patients with *ASPA* gene mutation(s) and a rescued iPSC line (CD#68 ASPA) with the wildtype (WT) *ASPA* gene knocked into the AAVS1 safe harbor site in CD#68 iPSCs generated in our previous study.^30^ The CD#68 ASPA iPSCs exhibited potent ASPA activity both *in vitro* and in transplanted CD mouse brains.^30^ We generated myelin spheroids from CD#59 and CD#68 iPSCs and CD#68 ASPA iPSCs. The ASPA activity of the CD#68 and CD#59 myelin spheroids was not detectable (ND), whereas the CD#68 ASPA spheroids that carry the WT *ASPA* transgene exhibited ASPA activity (54.17 nmol/mg/h) (Figure 5C). Because we changed medium for myelin spheroids every other day after week 6, NAA produced by neurons in the spheroids was not able to be accumulated in the *in vitro* culture system. Myelin spheroids generated from CD#68 or CD#59 iPSCs produced myelin sheaths normally (Figures 5A and S4G–J), even though CD#68 and CD#59 iPSCs carried a mutant *ASPA* gene. Accordingly, electron microscopy analysis revealed the ultrastructure of normal myelin sheaths from the CD#68 spheroid (Figure 5B). This result indicates that the *ASPA* mutation does not cause demyelination in the absence of NAA accumulation.

**Figure 6:**
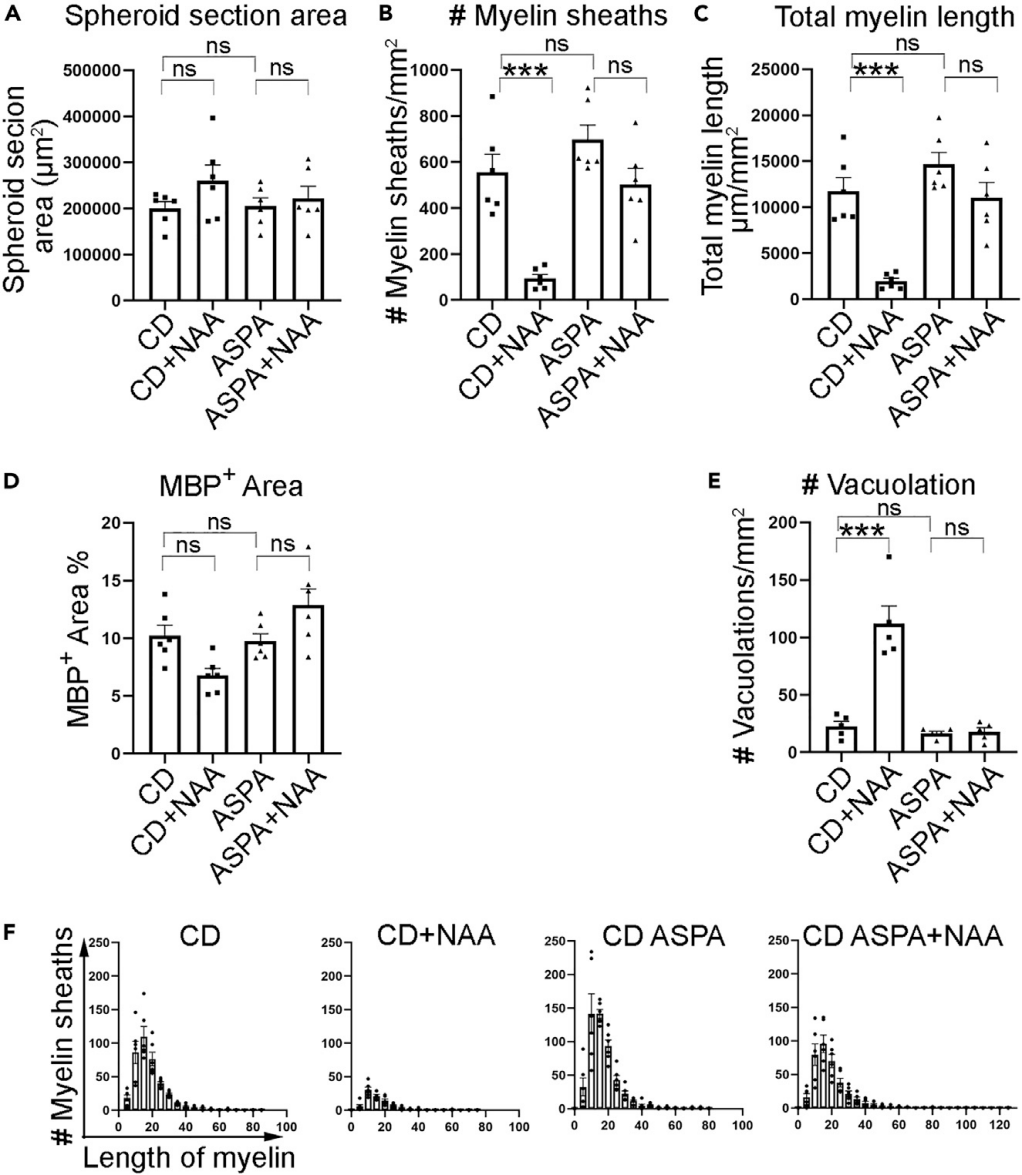
NAA disrupts myelin sheaths in CD patient iPSC-derived myelin spheroids (A) The area of spheroid sections is comparable in CD spheroids and ASPA spheroids with or without NAA treatment. n = 6 spheroids for each group. (B and C) The number (#) and total length of myelin sheaths were dramatically reduced in CD#68 spheroids, but not in CD#68 ASPA spheroids after NAA treatment. (D) The percentage of MBP^+^ area was not dramatically reduced in CD#68 or CD#68 ASPA spheroids after NAA treatment. (E) The number of vacuolation increased in CD#68 but not CD#68 ASPA spheroids after NAA treatment. (F) The distribution of the length of myelin sheaths. The total number of myelin sheaths from each group in panel F is shown in panel B. n = 6 spheroids for each group in a-d. n = 5 spheroids for each group in panel E. The average data from 3 sections of one spheroid were taken and plotted as one dot. Error bars are SE of the mean. ns: not statistically significant. ∗∗∗p < 0.001 by one-way ANOVA followed by Tukey’s multiple comparisons test for panels A–E.

The concentration of NAA in the brain of CD patients is about 8 mM or more.^41^^,^^42^ We tested NAA concentrations of 5 mM and 10 mM in the spheroids, and both concentrations induced effect. We chose 5 mM NAA for the following experiments. The spheroids were treated with NAA from week 16 to week 18 post-differentiation because we could start to see clear myelin sheaths in the spheroids around this stage. We treated myelin spheroids derived from CD#68 iPSCs or CD#68 ASPA iPSCs with 5 mM NAA for two weeks. After treatment, the spheroids derived from both iPSC lines exhibited no obvious morphological changes, with similar spheroid section areas (Figures 6A, S5A, and S5D). Immunostaining of sectioned spheroids revealed that both neurons (NeuN^+^ and Map2^+^) and astrocytes (SOX9^+^ and GFAP^+^) displayed no obvious change in morphology (Figures S5A and S5C) and cell density upon NAA treatment (Figures S5B and S5D). The cell density of oligodendroglial cells (OLIG2^+^) did not exhibit statistically significant decrease after NAA treatment either (Figure S5E). However, myelin sheaths were dramatically destroyed in CD#68 iPSC-derived myelin spheroids following NAA treatment (Figures 5A and 5B) as revealed by the dramatically reduced number and total length of myelin sheaths (Figures 6B and 6C). The number of vacuolation also increased in CD#68 myelin spheroids following NAA treatment (Figure 6E). Myelin sheaths from CD#59 myelin spheroids were also destroyed after NAA treatment (Figures S4G, S4I, and S4J), although the spheroids exhibited no overall morphological changes (Figure S4H). The MBP^+^ area was slightly decreased in CD#68 myelin spheroids following NAA treatment, but the decrease did not reach statistical significance (Figure 6D).

The structure of the myelin sheaths was disrupted substantially (Figures 5A and S4G). Scattered and swollen myelin sheaths were detected in CD#68 or CD#59 myelin spheroids after NAA treatment (Figures 5A and S4G). The vacuolized myelin sheath was also revealed by EM in CD#68 spheroids after NAA treatment (Figure 5B). A similar disruption of the myelin structure (Figure S6) was observed in the CD (nur7) mouse model carrying a mutant *ASPA* gene.^43^

In contrast, there is no obvious change in the myelin structure (Figures 5A and 5B), the number and total length of myelin sheaths, and the percentage of MBP^+^ area (Figures 6B–6D), and no increase in damaged myelin sheaths (Figure 6E) in CD#68 ASPA iPSC-derived spheroids upon NAA treatment. The experiments were repeated in two additional batches of CD#68 and CD#68 ASPA spheroids that were derived independently, and similar results were obtained (Figure S5).

The concentration of NAA inside the CD#68 spheroids was increased substantially after NAA treatment (from 0.715 μmol/g to 1.562 μmol/g), whereas the NAA level was not detectable (ND) in CD#68 ASPA spheroids that carry the WT ASPA enzyme either with or without exogenous NAA treatment (Figure 5D). The undetectable NAA level in CD#68 ASPA spheroids even after NAA treatment suggests that the functional ASPA enzyme in the CD#68 ASPA spheroids can catalyze the conversion of NAA to maintain low level of NAA in the spheroids, thus preventing high-level NAA-induced myelin toxicity. There was no dramatic change of acetate level in the spheroids after NAA treatment (Figure 5D). These results indicate that high-level NAA can induce myelin defects when the *ASPA* gene is mutated, and that the introduction of a WT *ASPA* gene can prevent NAA-induced myelin toxicity.

## Discussion

In this study, we developed a simple and highly efficient protocol to generate myelinated spheroids from hiPSCs following the developmental process of oligodendrocyte genesis and myelin formation. This protocol allowed us to detect early myelination starting from week 12 (day 84) after differentiation, sooner than day 105 to day 140 in previous reports.^21^^,^^22^^,^^26^ Importantly, compact myelin sheaths with paranodes can be detected in our spheroids. Using this protocol, we derived myelin spheroids from CD patient iPSCs, which could recapitulate key pathological features of the disease, including deficiency in ASPA activity and demyelination.

The limitation of two-dimensional (2D) culture system to replicate human brain pathologies in a 3D cellular context has triggered the generation of 3D culture system such as organoids or spheroids.^9^^,^^11^^,^^12^^,^^13^^,^^15^^,^^44^ It is difficult to create comprehensive multi-layered myelin sheaths, highly specialized membrane structures, in a 2D culture system. What is especially difficult to achieve in a 2D system is myelin organization that requires long-term culture within a stable environment. Human OPCs and oligodendrocytes have been derived in the 2D system by different protocols.^33^^,^^38^^,^^45^^,^^46^^,^^47^^,^^48^ However, fully organized myelin structure has yet to be generated in the 2D system. While several methods have been developed to generate 3D spheroids with myelination,^20^^,^^21^^,^^22^^,^^26^^,^^49^ there are limitations including either the lack of widespread compact myelin sheaths or the need of long-term differentiation. For example, compact myelin sheaths with paranodes were not observed in some of the spheroids reported earlier.^21^^,^^22^ A recently developed protocol can produce spheroids with compact myelination by using insert wells to create the air-liquid interface,^26^ however, handling the air-liquid interface can be technically challenging. Here, we have developed a protocol to generate myelin spheroids containing compact myelin sheaths with paranodes in flasks. A large number of spheroids with consistent quality can be generated in flasks. The handling is straightforward. Medium change in flasks is standard. No special device or technique is needed. Moreover, our method is developed based on a widely-used oligodendrocyte differentiation protocol^32^^,^^33^ that has been used in multiple studies in different laboratories and proven easy to handle and can yield reproducible differentiation.^30^^,^^38^^,^^50^^,^^51^^,^^52^^,^^53^^,^^54^ We modified this protocol by continued culturing of oligospheres in a suspension system instead of attaching the oligospheres for 2D differentiation. This study suggests that OPC or oligodendrocyte differentiation protocols can be used to generate myelin spheroids by switching attached cultures to suspension cultures.

According to the primary defect of myelin, myelin disorders can be classified into three categories: hypomyelinating (i.e., lack of myelin deposition), demyelinating (i.e., loss of previously deposited myelin), and dysmyelinating (i.e., deposition of structurally or biochemically abnormal myelin).^55^ Compared to the 2D culture system, myelin spheroids are advantageous for modeling demyelination and dysmyelination diseases, because myelin spheroids allow the generation of widespread myelin sheaths, which are needed as a control to show loss of myelin sheaths under disease conditions. In this study, we generated myelin spheroids from CD patient iPSCs and used these spheroids to model CD, a demyelinating disease. We were able to recapitulate disrupted myelin structure, the most common pathologic alteration of myelin sheaths in myelination diseases,^1^ in NAA-treated CD patient iPSC-derived myelin spheroids, a human cellular model established for this disease for the first time. Moreover, introducing the WT *ASPA* gene was able to rescue NAA-induced myelination defects in the spheroids.

There have been long-time debates about CD pathological mechanisms. It remains to be determined whether the supraphysiological level NAA-induced toxicity or the undersupply of NAA-derived acetate directly causes demyelination.^1^^,^^56^^,^^57^ The CD mouse models exhibit both accumulation of NAA and reduction of acetate level,^58^ which makes it difficult to tease out which aspect is key to CD pathological manifestations. Recently, studies using the *Natl8*/*Aspa* double knockout mice showed that ablating NAA synthesis prevented development of vacuolation,^59^^,^^60^^,^^61^ suggesting that NAA toxicity may be a key factor responsible for demyelination. However, the short life span of the *Nat8l* knockout mice and the *Natl8*/*Aspa* double knockout mice suggests that NAT8L is critical for development, thus complicating the interpretation related to the NAA toxicity.^60^^,^^62^ By using an *in vitro* myelin spheroid model, we provided direct evidence to demonstrate NAA toxicity on myelin sheaths in a 3D human cellular platform. We found that spheroids derived from CD patient iPSCs that carry a mutant *ASPA* gene could form myelin sheaths normally in a culture system with constant medium change to avoid NAA accumulation. However, the CD spheroids treated with NAA exhibited dramatically reduced myelin sheaths. Thus, this study provides compelling evidence that myelination defects in CD can be caused by NAA toxicity, although it does not exclude the possibility that the deficiency of acetate may also contribute to demyelination. Moreover, using myelin spheroids derived from ASPA iPSCs that were introduced with the WT *ASPA* gene, we have shown that myelin sheaths generated from oligodendrocytes carrying the WT *ASPA* gene can resist the toxicity from treatment with NAA, supporting the idea of therapeutic development by introducing a functional *ASPA* gene into CD patients through gene therapy^61^^,^^63^^,^^64^ or combined cell and gene therapy.^30^^,^^65^

Collectively, we have developed an easy-to-follow and reproducible protocol to generate spheroids with compact myelin sheaths and used the myelin spheroids to model CD, a demyelinating disease. This 3D human cellular model has allowed us to recapitulate key pathological features of the disease and provide evidence to support NAA-induced myelin toxicity, which helps to resolve long-term debates about the pathological mechanism of the disease. The CD spheroid model also allowed us to demonstrate that the WT *ASPA* gene was able to rescue CD disease phenotypes in a human cellular model, thus introducing a functional *ASPA* gene can be used as an effective therapeutic strategy for treating CD patients. The myelin spheroid platform we have developed in this study provides a powerful platform for studying myelin function in health and diseases, including not only leukodystrophies but also MS and other neurological disorders with myelin defects.

### Limitations of the study

In this study, we have developed a protocol to generate myelin spheroids containing compact myelin sheaths with paranodes in a simplified and standard manner. This protocol allowed us to detect early myelination starting from week 12 after differentiation. However, it is still a lengthy process. Further optimization to shorten the process is a future direction.

## STAR★Methods

### Key resources table

**Table:** 

REAGENT or RESOURCE	SOURCE	IDENTIFIER
**Antibodies**		

Mouse monoclonal anti-CASPR	Millipore Sigma	Cat# MABN69; RRID: AB_10806491
Mouse monoclonal anti-CC1 (APC)	Millipore Sigma	Cat# MABC200; RRID: AB_11203645
Rabbit polyclonal anti-NF200	Millipore Sigma	Cat# N4142; RRID: AB_477272
Mouse monoclonal anti-MOG	Millipore Sigma	Cat# MAB5680; RRID: AB_1587278
Mouse monoclonal anti-MAG	Millipore Sigma	Cat# MAB1567A4
Rat monocolonal anti-MBP	Millipore Sigma	Cat# MAB386; RRID: AB_94975
Chicken polyclonal anti-Map2	Abcam	Cat# ab5392; RRID: AB_2138153
Rabbit polyclonal anti-OLIG2	Millipore Sigma	Cat# AB9610; RRID: AB_570666
Rabbit polyclonal anti-GFAP	Agilent (Dako)	Cat# Z033429-2; RRID: AB_10013382
Goat polyclonal anti-SOX9	R&D	Cat# AF3075; RRID: AB_2194160
Goat polyclonal anti-SOX10	R&D	Cat# AF2864; RRID: AB_442208
Rabbit polyclonal anti-NEUN	GeneTex	Cat# GTX16208
Rabbit polyclonal anti-Cleaved Caspse-3	Cell Signaling	Cat# 9661; RRID: AB_2341188

**Chemicals, peptides, and recombinant proteins**		

E8	ThermoFisher	Cat# 15169-01
DMEM/F12	ThermoFisher	Cat# 11330057
GlutaMAX	ThermoFisher	Cat# 35050-061
NEAA	ThermoFisher	Cat# 11140-050
Insulin	Millipore Sigma	Cat# I9278
N2	ThermoFisher	Cat# 17502048
B27-VA	ThermoFisher	Cat# 12587010
RA	Millipore Sigma	Cat# R2625
SB431542	Peprocell	Cat# 04-0010-10
LDN-193189	Peprocell	Cat# 04-0074-10
SAG	Millipore Sigma	Cat# SML1314
T3	Millipore Sigma	Cat# T2877
Biotin	Millipore Sigma	Cat# B4639
dbcAMP	Millipore Sigma	Cat# D0627
PDGF	R&D	Cat# 221-AA-100
IGF-1	R&D	Cat# 291-G1-200
HGF	R&D	Cat# 294-HG-250
NT3	Peprotech	Cat# AF-450-03
Ascorbic acid	Millipore Sigma	Cat# A4403
NAA	Millipore Sigma	Cat# 00920

**Experimental models: Cell lines**		

I90(IMR90) fibroblast	Coriell	I90-10
CD#59 fibroblast	Coriell	GM00059
CD#68 fibroblast	Coriell	GM04268
I90 iPSC	The Shi Lab	NA
CD#59 iPSC	The Shi Lab	NA
CD#68 iPSC	The Shi Lab	NA
CD#68 ASPA iPSC	The Shi Lab	NA

**Experimental models: Organisms/strains**		

ASPA^*nur7*^/J mice	Jackson Laboratory	008607

**Software and algorithms**		

Photoshop	Adobe	N/A
Zen 2.3 lite	Zeiss	N/A
Image-Pro Premier 9.2	Media Cybernetics	N/A
Nikon Ti-2	Nikon	N/A

### Resource availability

#### Lead contact

Further information and requests for resources and reagents should be directed to and will be fulfilled by the lead contact, Yanhong Shi (yshi@coh.org).

#### Materials availability

All unique reagents generated in this study are available from the lead contact with a completed materials transfer agreement.

### Experimental model and subject details

#### Cell lines

I90 iPSCs were generated from I90 fibroblasts (IMR90, female, 16-week gestation, Coriell) and characterized in our previously study.^38^ CD#59 and CD#68 iPSCs were derived from CD patient fibroblasts GM00059 (female, 1-year-old, Coriell) and GM04268 (male, 2-year-old, Coriell), respectively. The CD#59 iPSCs carry the G176E and A305E mutations and a Y231X polymorphism in the *ASPA* gene and CD#68 iPSCs carry the E285A mutation in the *ASPA* gene. The CD#68 ASPA iPSCs were derived by introducing the WT *ASPA* gene under the EF1α promoter into CD#68 iPSCs through transcription activator-like effector nuclease (TALEN)-mediated gene-editing as described in our previous study.^30^ Both I90 and CD iPSCs have been authenticated by STAR assay and lack of mycoplasma contamination.

#### Generation and maintenance of CD (Nur7) mice

All animal housing conditions and procedures were approved by and conducted according to the Institutional Animal Care and Use Committee of City of Hope. CD (Nur7) mice (ASPAnur7/J, 008607) were purchased from the Jackson Laboratory.^43^ The CD phenotypes in this mouse model have been fully characterized in previous studies.^30^ Brains were collected from a 3-month-old homozygous (male) and a 3-month-old heterozygous mouse (female). Brain sections were stained for MBP to show damaged myelin sheaths.

### Method details

#### Generation of myelin spheroids

The myelin spheroids protocol was developed based on a previously published protocol.^33^^,^^38^ The medium composition at each step is listed in Table S1. Briefly, iPSCs were dissociated into single cells, seeded onto Matrigel-coated plates and induced by medium (M)-I (M-I) for 8 days. Then cells were switched to M-II for another 4 days to induce cells to the glial lineage. After 12 days of culture, about 1 × 10^7^ cells were dissociated and cultured in a T75 suspension flask without coating to form spheroids. The resultant spheroids were switched to M-III for 8 days. On day 20, M-IV with growth factors including PDGF was added to induce OPC formation and expansion for 10 days. The cells were cultured without shaking to allow cells to form spheres spontaneously. From day 30, the flask was put on a shaker at 50 rpm rotating speed and medium was switched to M-V for oligodendrocyte generation and myelination. Roughly 75% of the medium was changed every 48 hours. Spheroids were collected every two weeks and stained for OPC and oligodendrocyte markers. The size of the spheroids is usually between 200 μm to 1000 μm in diameter.

#### NAA treatment of myelin spheroids

Myelin spheroids were generated from CD#59 iPSCs, CD#68 iPSCs or CD#68 ASPA iPSCs and treated with NAA after myelin sheaths were formed in spheroids. To prepare NAA treatment medium, NAA was added to glia medium (M-V medium) to 5 mM final concentration and the pH was adjusted back to 7 by NaOH solution. The spheroids from same line were cultured in one flask before treatment. At week 16, 20–30 spheroids were transferred into T-75 flask in 20 ml glial medium with or without 5 mM NAA. 90% of the medium with 5 mM NAA or control medium without NAA was changed every 48 hours. After two-week treatment, spheroids were collected for NAA level measurement. The spheroids from three different differentiation batches were treated in three independent experiments.

#### ASPA enzymatic activity assay

The ASPA enzymatic activity assay was performed as described previously.^30^ Briefly, spheroids were dissociated with accutase. Cell lysates were prepared using RIPA buffer with PMSF and protein concentration was determined by Bradford. Then 100 μg protein lysates were used for two-steps reaction and OD340 nm was determined by luminescence reader. The ASPA activity is defined by the production of aspartate in nmol by 1 mg protein lysate in 1 hr at 37°C.

#### NAA level measurement

To measure the concentration of NAA in spheroids, the spheroids with or without NAA treatment were collected and washed with DPBS. The weight of spheroids was measured. Then the spheroids with or without NAA treatment were centrifuged at 300 g for 3 min. The spheroid pellets were sonicated to lyse cells. After sonication, tubes containing the lysed spheroids were incubated in boiling water for 3 min to deactivate enzymes and then centrifuged at 12,000 rpm for 10 min. The supernatant was collected and added with 300 μL of 50 mM NaPO4 buffer. Samples were then subjected to NMR analysis at the NMR Core Facility of City of Hope.

#### Immunohistochemistry

Spheroids were fixed in 4% PFA for 1 hr and submerged in 30% source at 4 degree for overnight. After sinking to the bottom of the container, spheroids were embedded in the OCT compound and serially sectioned at 14 μm thickness using Lecia CM3050S. Specifically, slides were first labeled. Serial sections were collected onto labeled slides with one section per slide, until all slides were used for collection. The procedure was repeated until all sections from one spheroid were collected. 3-4 sections from different positions of one spheroid were put onto one slide. Mouse brains were perfused and post-fixed in 4% PFA, then cryoprotected with 30% sucrose. Cryoprotected brains were flash frozen and serially sectioned at sagittal planes.

For immunohistochemistry analysis, spheroid or brain sections were permeabilized in PBS with 0.1% Triton X-100 (PBST) for 3 × 5 min, blocked with 5% donkey serum in PBST for 1 hour at RT. Sections were then incubated with primary antibodies (Table S1) at 4°C for overnight. Following primary antibody incubation and washes, sections were incubated with secondary antibodies at RT for 1 h, washed with 1 x PBS, counterstained with Dapi, and mounted with the mounting medium.

For spheroid imaging, the image of each whole section was taken. The whole section images of spheroids and tiled images of mouse cerebellum were taken with Nikon Ti-2. Confocal imaging was performed on a Zeiss LSM 700 microscope (Zeiss), and the resulting images were analyzed with Zen 2.3 lite software (Zeiss). For z-stack imaging, we used 1 μm per step. Images were organized using Photoshop. Schematic images were created by BioRender.com.

For quantification, sequential sections on one slide were used for quantification, except for Caspr quantification. Caspr quantification was performed using confocal images: one confocal image was taken randomly from each section and three images were taken from three sections of one spheroid for Caspr quantification.

#### Transmission electron microscopy (TEM) and immunogold staining

Spheroids were fixed with 2.5% glutaraldehyde, 0.1 M cacodylate buffer (Na(CH3)2AsO2 ·3H2O), pH7.2, at 4°C. Standard sample preparation for TEM was followed including post-fixation with osmium tetroxide, serial dehydration with ethanol, and embedding in Eponate.^66^ Ultra-thin sections (70 nm thick) were acquired by ultramicrotomy, post-stained, and examined on FEI Tecnai 12 transmission electron microscope equipped with a Gatan Ultrascan 2K CCD camera. Immunogold staining was performed using the MAG antibody (Table S1) and Nanogold® anti-rabbit IgG (Nanoprobes). HQ Silver enhancement kit (Nanoprobes) was used to enlarge the Nanogold® staining. Spheroids were processed following standard sample preparation for TEM and imaged.

### Quantification and statistical analysis

For quantification of fluorescence images, three sections of each spheroid were imaged using Nikon Ti-2 for the whole section. The area of sections was measured by ImagePro and Nikon NIS-Elements. Olig2, SOX10, MBP, cleaved Caspase-3, Map2, SOX9, and NeuN-positive cells in the whole section were manually counted using Photoshop. DAPI-positive cells were counted using Qupath software.^67^ The myelin sheaths stained by MBP were manually traced and the number and length of myelin sheaths was measured by Nikon NIS-Elements. For quantification of Caspr-positive myelin sheaths, one image from one section, total three images from each spheroid were taken using Zeiss LSM 700 confocal (Zeiss). The graphs were made using GraphPad Prism 8 (San Diego, CA). The average data from three sections of one organoid were taken and used as one biological replicate and plotted as one dot in the graph.

For g-ratio quantification of TEM images, the inner axon diameter and the outer diameter of a myelin sheath were measured by Image-Pro 9.1. The graphs were made using GraphPad Prism 8. The g-ratio of one myelin sheath is shown as one dot in the graph. Three spheroids were analyzed in each group.

Data are shown as means ± SE as specified in the figure legends and analyzed using GraphPad Prism 8 (San Diego, CA). The number of spheroids analyzed per group is indicated as “n” in the corresponding figure legends. Spheroids were assigned randomly to treatment groups. The study was not blinded. One-Way ANOVA followed by Tukey’s multiple comparisons test or Student’s *t* test (two tailed) was used for statistical analysis as reported in each figure legend. p < 0.05 was considered statistically significant. ∗p < 0.05, ∗∗p < 0.01 and ∗∗∗p < 0.001.

## Data Availability

• All data reported in this paper is available within the paper and the supplemental information. • This paper does not report original code. • Any additional information required to reanalyze the data reported in this paper is available from the lead contact upon reasonable request.
