# Supplementary material for: Developing a human iPSC-derived three-dimensional myelin spheroid platform for modeling myelin diseases
Source: iScience. 2023 Sep 25;26(11):108037. doi: 10.1016/j.isci.2023.108037 (PMC10589867; doi:10.1016/j.isci.2023.108037)
Supplement: Document S1. Figures S1–S6 and Table S1 [file mmc1.pdf]

iScience, Volume *26*

Supplemental information

Developing a human iPSC-derived three-dimensional myelin spheroid platform for modeling myelin diseases

Lizhao Feng, Jianfei Chao, Mingzi Zhang, Elizabeth Pacquing, Weidong Hu, and Yanhong Shi

Supplemental Table

Supplemental Table S1 The medium composition at each step, related to STAR METHODS "Generation of myelin spheroids".

Step	Day	Medium
**Step 1** Attachment	Day 0-7 M-I	Basal media* + 1x N2 + 0.1μM RA + 10 μM SB431542 + 250 nM LDN-193189
	Day 8-11 M-II	Basal media + 1x N2 + 0.1μM RA + 1 μM SAG
**Step 2** Suspension	Day 12-19 M-III	Basal media + 1x N2 + 0.1μM RA + 1 μM SAG + 1x B27-VA
	Day 20-29 M-IV	Basal media + 1x N2 + 1x B27-V A + 60 ng/ml T3 + 100 ng/ml Biotin + 1 μM dbcAMP + 10 ng/ml PDGF +10 ng/ml IGF-1 + 5 ng/ml HGF + 10 ng/ml NT3
	>Day 30 M-V	Basal media + 1x N2 + 1x B27-VA + 60 ng/ml T3 + 100 ng/ml Biotin + 1 μM dbcAMP + 20 μg/ml Ascorbic acid

*Basal media: DMEM/F12, 1x GlutaMAX, 1x NEAA, 25 μg/ml Insulin

Supplemental Figures and Legends

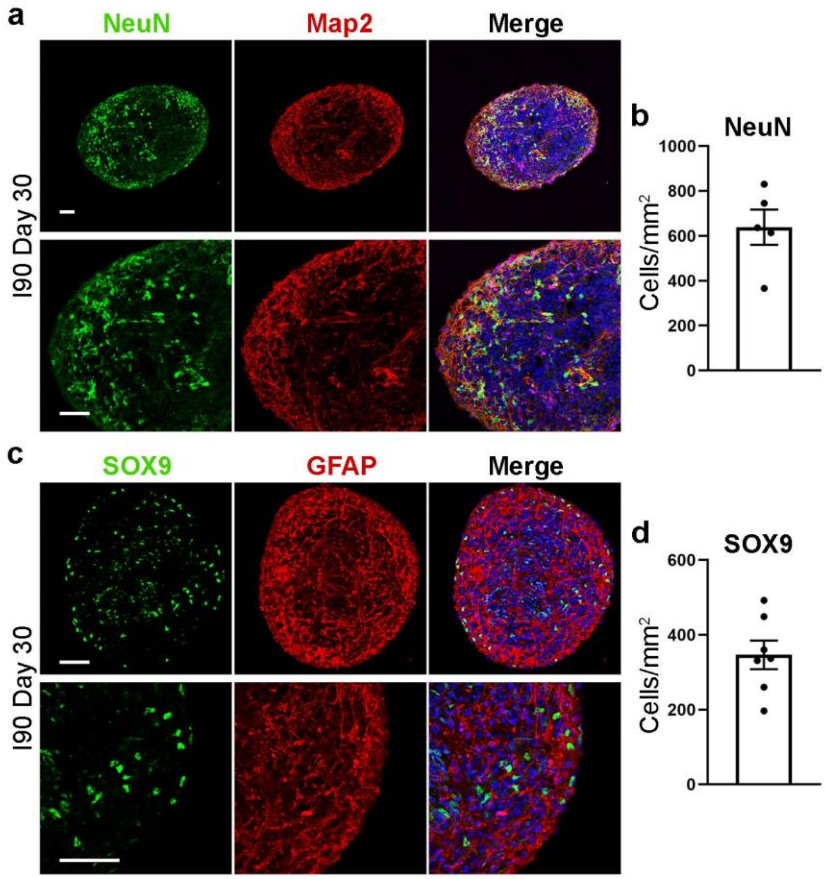

Figure S1 Myelin spheroids contain neurons and astrocytes as early as day 30 after differentiation, related to Figure 1. a, Immunostaining for mature neuron markers NeuN and MAP2. Scale bar: 50 µm. **b**, Quantification of $NeuN^+$ neurons in I90 spheroids. n=5 spheroids. **c**, Immunostaining for astrocyte markers SOX9 and GFAP. Scale bar: 50 µm. **d**, Quantification of $SOX9^+$ astrocytes in I90 spheroids. n=7 spheroids. The average data from 3 sections of one spheroid were taken and plotted as one dot.

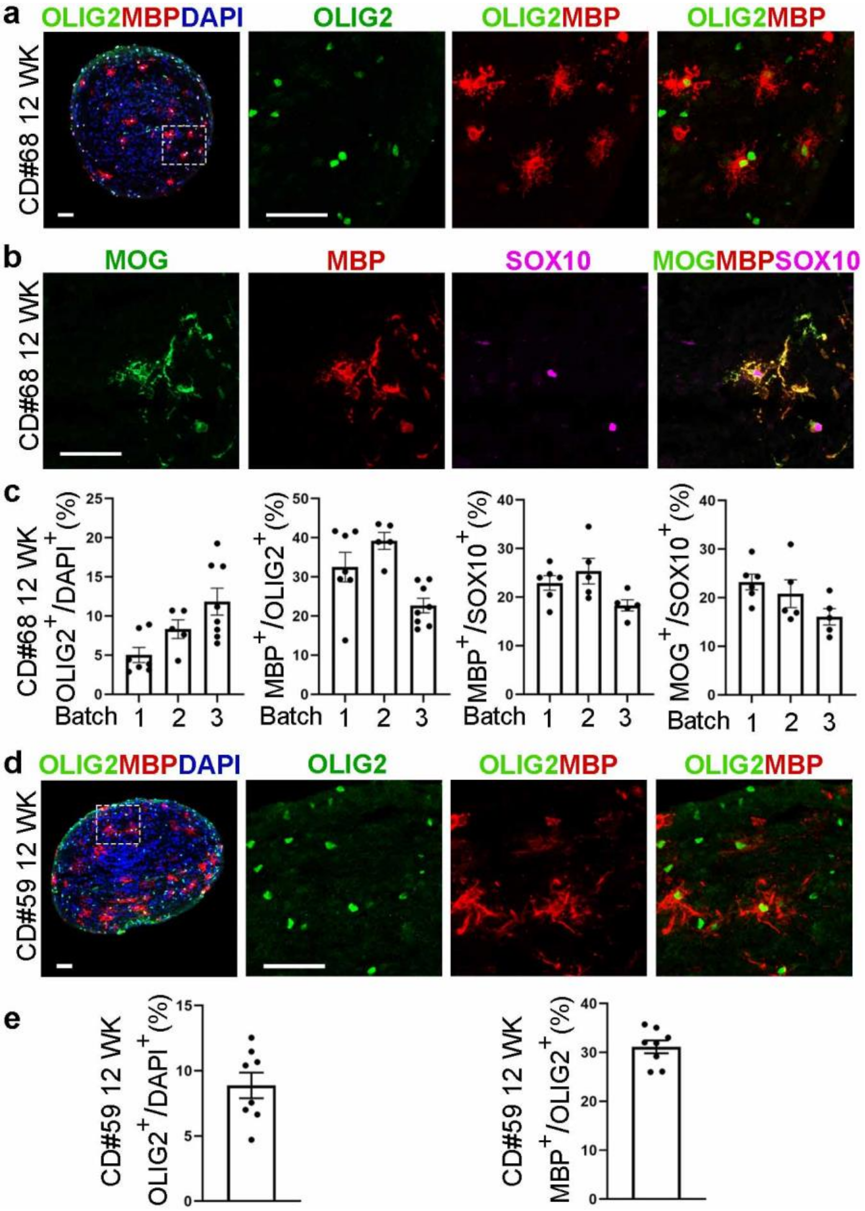

Figure S2 Characterization of CD#68 and CD#59 iPSCs-derived oligodendroglial spheroids, related to Figure 1. **a**, Spheroids generated from CD#68 iPSCs co-expressed the oligodendeoglial lineage marker OLIG2 and the oligodendrocyte marker MBP. **b**, Spheroids generated from CD#68 iPSCs co-expressed the oligodendeoglial lineage marker SOX10 and the oligodendrocyte markers MBP and MOG. **c**, The percentage of the OLIG2^{+} oligodendroglial cells, the MBP^{+} oligodendrocytes (MBP$^{+/}$OLIG2^{+} and MBP^{+}/SOX10^{+}), and the MOG^{+} oligodendrocytes (MOG^{+}/SOX10^{+}) in three batches of CD#68 spheroids on week 12. $n \geq 5$ spheroids for each group. **d**, Spheroids generated from CD#59 iPSCs co-expressed the oligodendroglial lineage marker OLIG2 and the oligodendrocyte marker MBP. **e**, The percentage of the OLIG2^{+} oligodendroglial lineage cells and the MBP^{+} oligodendrocytes (MBP^{+}/OLIG2^{+}) in CD#59 spheroids on week 12. n=8 spheroids. The average data of 3 sections from each spheroid were taken and plotted as one dot.

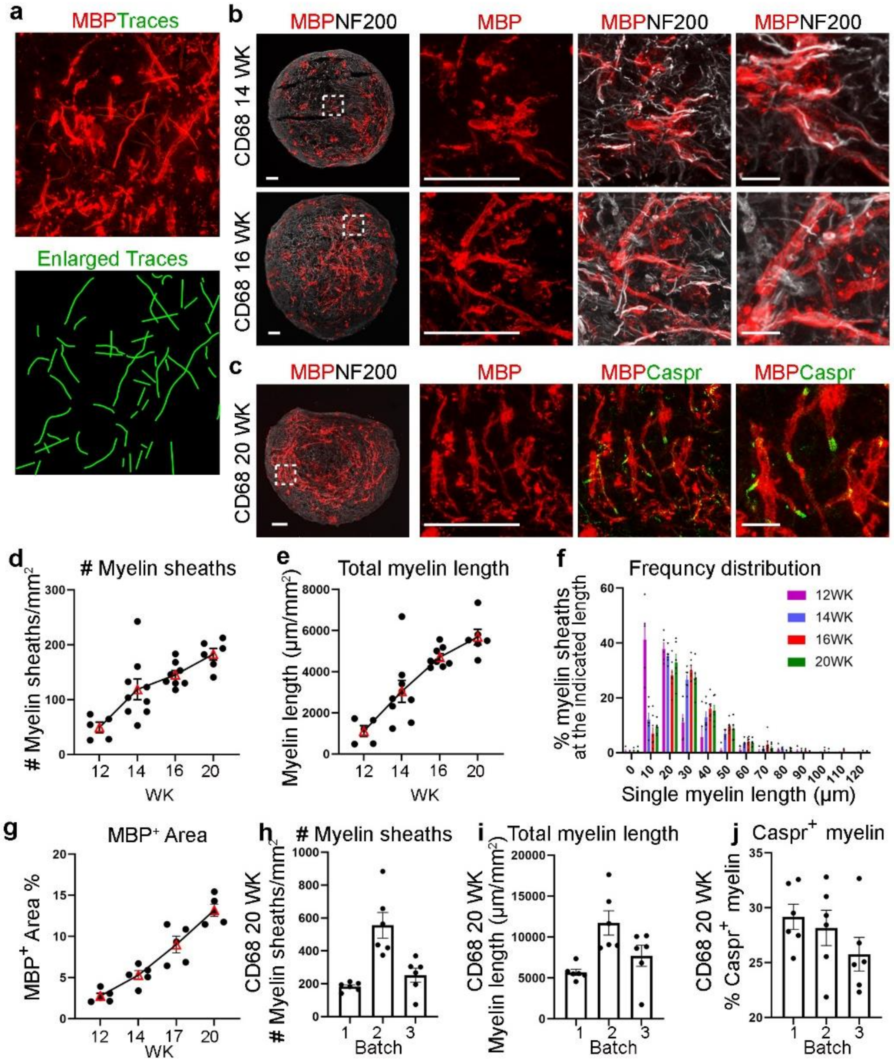

Figure S3 Myelin formation in myelin spheroids derived from CD#68 iPSCs, related to Figure 2 and Figure 3. **a**, The original traces generated by the software are shown together with the MBP signal in the left panel. The traces from the left panel are thickened and shown in the right panel for easier visualization. b. Immunostaining of myelin sheaths for MBP and NF200 in CD#68 myelin spheroids at week 14 and week 16. Scale bar: 50 µm for the left four panels; 10 µm for the rightmost panel. **c**, Immunostaining of MBP and the paranodal marker Caspr in CD#68 myelin spheroids at week 20. Scale bar: 50 µm for the left four panels; 10 µm for the right most panel. **d**, **e**, The number and total length of the MBP^+ myelin sheaths increased in spheroids from week 12 to week 20. n=5 spheroids for week 12, n=9 spheroids for week 14, n=8 spheroids for week 16, and n=6 spheroids for week 20. **f**, The frequency distribution of the MBP^+ single myelin sheath length from week 12 to week 20. n=128 myelin sheaths from 5 spheroids for week 12, n=691 myelin sheaths from 9 spheroids for week 14, n=793 myelin sheaths from 8 spheroids for week 16, and n=677 myelin sheaths from 6 spheroids for week 20. **g,** The percentage of MBP^+ area increased in spheroids from week 12 to week 20. n=5 spheroids for each group. **h**, **i**, Quantification of the number and total length of myelin sheaths in 3 batches of CD#68 spheroids on week 20. n=6 spheroids for each batch. The data from week 12 in panels **d** and **e** are shown as batch 1 result in panels **h** and **i**, respectively. **j**, The percentage of the $Caspr^+$ myelin sheaths in 3 batches of CD#68 spheroids on week 20. n=6 spheroids for each batch. The average data of 3 sections from each spheroid were taken and plotted as one dot for panel **d**, **e, g, h, i** and **j**.

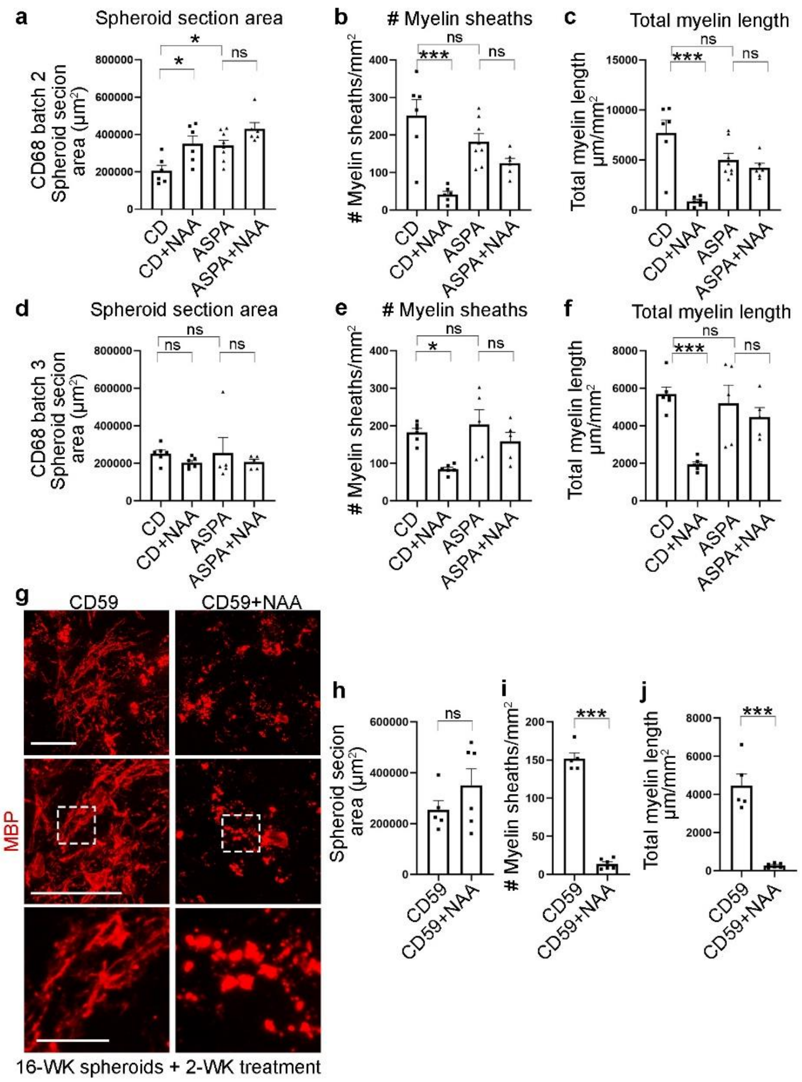

Figure S4 NAA treatment reduces the number of myelin sheaths and the total myelin length in CD#68 spheroids in multiple batches and in CD59# spheroids, related to Figure 5 and 6. Batch 1 results are shown in Figure 6 and batch 2 and batch 3 results are shown here for CD#68 spheroids in panels a-f. **a**, **d**, The area of spheroid sections is comparable in CD#68 spheroids and ASPA spheroids with or without NAA treatment. n= 6 spheroids for each group. **b, c, e** and **f**, The number and total length of myelin sheaths were dramatically reduced in CD#68 spheroids after NAA treatment. The results from 2 batches of spheroids are shown here. n=6 spheroids for groups (CD, CD+NAA and ASPA+NAA) and n=8 spheroids for the ASPA group (ASPA) in batch 2. n=6 spheroids for groups (CD and CD+NAA) and n=5 spheroids for groups (ASPA and ASPA+NAA) in batch 3. g, Immunostaining of CD#59 myelin spheroids with or without NAA treatment for MBP. CD#59 hiPSC-derived spheroids formed myelin sheaths normally in vitro. Myelin damage was observed in CD#59 spheroids after NAA treatment. Images were enlarged sequentially from the top to the bottom. Scale bar: 50 μm for the top and middle rows; 10 μm for the bottom row. **h**, The section area of the spheroids are shown. **i**, **j**, The number and the total length of myelin sheaths were dramatically reduced in CD#59 spheroids after NAA treatment. n=5 spheroids for CD#59 group and n=6 spheroids for CD#59+NAA group. The average data of 3 sections from each spheroid were taken and plotted as one dot. Error bars are SE of the mean. ns: not statistically significant. *$p<0.05$ and ***$p<0.001$ by one-way ANOVA followed by Tukey's multiple comparisons test for all panels.

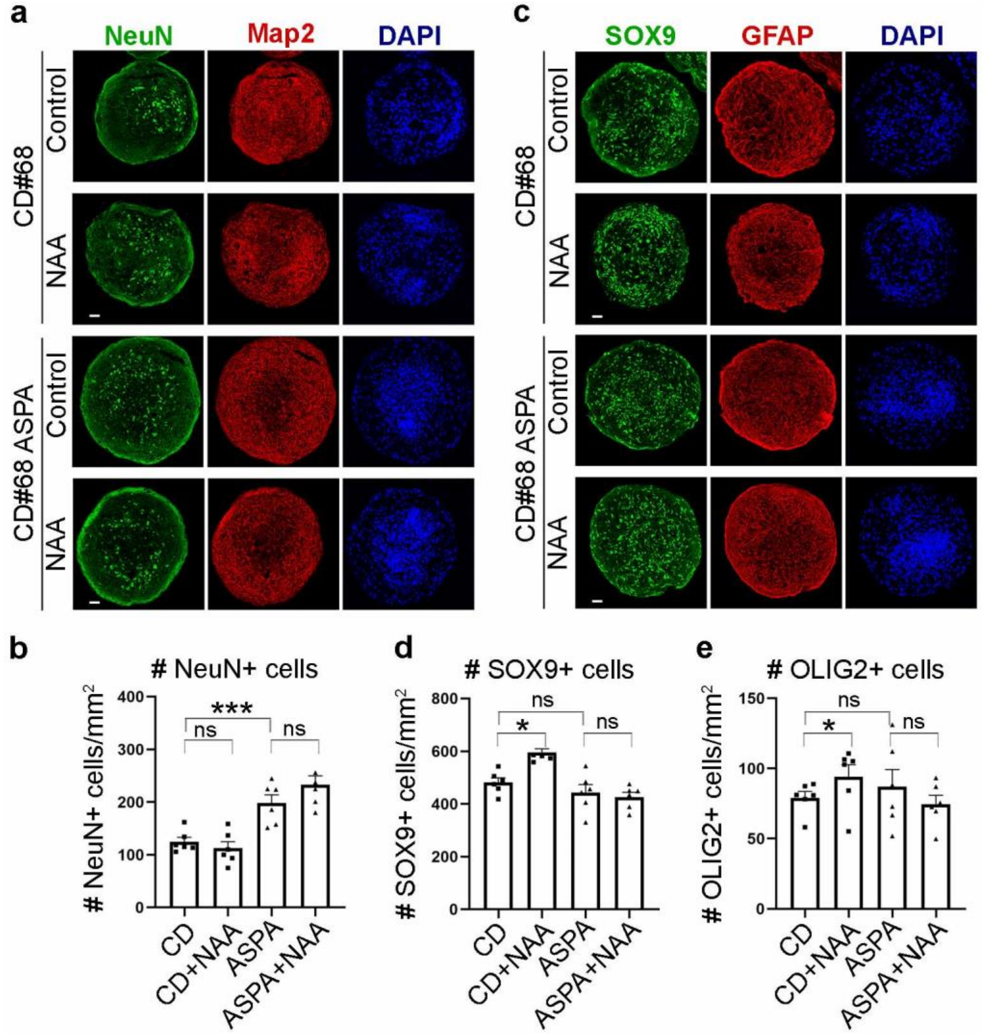

Figure S5 Neurons and astrocytes in CD myelin spheroids, related to Figure 5. a, Immunostaining of CD myelin spheroids with or without NAA treatment for neuronal markers NeuN and MAP2. The morphology of neurons displayed no obvious difference after NAA treatment. Scale bar: 50 µm. **b**, The number of $NeuN^+$ cells in myelin spheroids. **c**, Immunostaining of CD myelin spheroids with NAA treatment for astrocyte markers SOX9 and GFAP. The morphology of astrocytes displayed no obvious change after NAA treatment. Scale bar: 50 µm. **d**, The number of $SOX9^+$ cells in myelin spheroids. Increased density of astrocytes was observed in CD spheroids after NAA treatment. n= 6 spheroids for each group, the average data of 3 sections from each spheroid were taken and plotted as one dot for panels **b** and **d**. Error bars are SE of the mean. ns: not statistically significant. *p<0.05 and ***p<0.001 by one-way ANOVA followed by Tukey's multiple comparisons test for panels **b** and **d**.

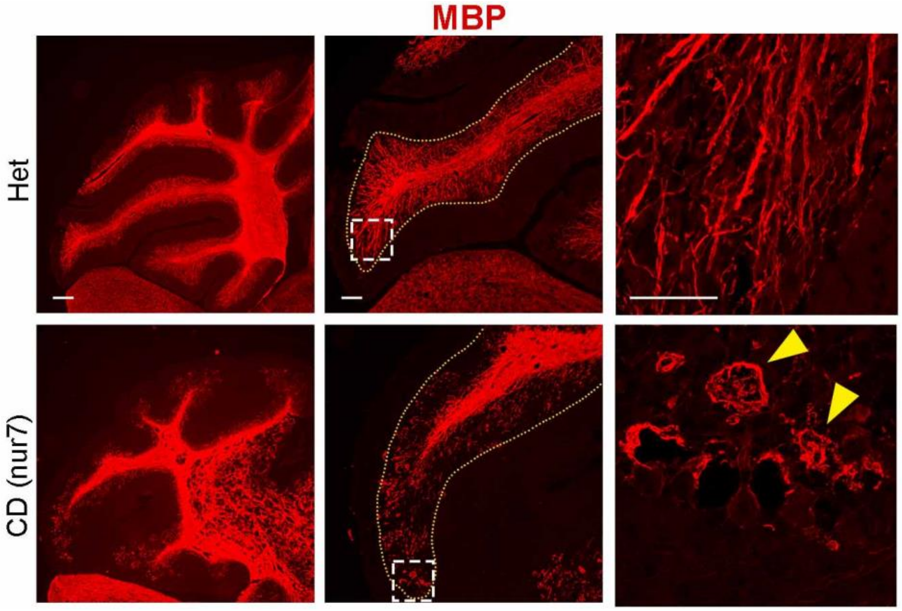

Figure S6 CD (nur7) mouse model shows myelin damage, related to Figure 5. Immunostaining of mouse brains for MBP in CD (nur7) heterozygous (upper panels) or homozygous (lower panels) mice at 3 months old. Images of cerebellum of the brain were enlarged sequentially from left to right. The CD (nur7) homozygous mouse showed vacuolation-like myelin damage and reduced density of myelin sheaths compared to the heterozygous control. Arrow heads point to vacuolation-like myelin damage. Scale bar: 250 µm for the left panels; 100 µm for the middle panels; and 50 µm for the right panels.
